# Study on Pulse-Reverse Electroplating Process for the Manufacturing of a Graphene-Based Coating

**DOI:** 10.3390/ma16020854

**Published:** 2023-01-16

**Authors:** Gabriele Baiocco, Silvio Genna, Erica Menna, Nadia Ucciardello

**Affiliations:** Department of Enterprise Engineering Mario Lucertini, University of Rome Tor Vergata, 00133 Rome, Italy

**Keywords:** pulse reverse electroplating, composite coatings, graphene, electrical conductivity, aluminum

## Abstract

This work investigates the feasibility of increasing the electric conductivity of an AA1370 aluminium wire by using pulse-reverse electrodeposition to realize Cu-Graphene composite coating. The graphene adopted was in the form of nanoplates (GnP). To study the effects of plating parameters, a 2^3^ factorial plan was developed and tested. During the tests, the following process parameters were varied: the current density, the frequency and the duty cycle. The ANalysis Of VAriance (ANOVA)) was adopted to evaluate their influence on the coated wires’ morphology and electrical conductivity resistance. The results show that all the tested conditions allow good compactness to the coating, and the amount of graphene is well incorporated within the microstructure of the copper deposit. In addition, in the best conditions, the electrical resistivity decreases up to 3.4% than the uncoated aluminum.

## 1. Introduction

Copper and copper alloys are used in many applications, such as electrical wire and wire connectors, heat exchanger pipes and marine structures. In addition, copper plating is employed in semiconductors and electronics to manufacture metal interconnects and many electronic devices such as laptops and mobile [1]. Therefore, copper is extensively used either because of its conductivity properties for heat and electrical charge transfer applications or to produce metallic films [2]. Aluminum has been applied as a copper substitute due to its good electrical conductivity and strength-to-weight ratio [3]. However, compared with copper, its lower electrical conductivity requires an increase in the conductor cross-section of 60% and the lower thermal conductivity limits the heat dissipation in case of hotspots generation [4]. Additionally, the low melting temperature does not allow it to withstand overloads for a long time. Therefore, improving the electrical and thermal conductivity of aluminum is an important issue. Graphene exhibits outstanding charge carrier mobility and thermal properties [5,6,7,8] in addition to remarkable mechanical properties (Young’s modulus = 1 TPa, intrinsic breaking strength = 130 GPa). These characteristics make graphene an appealing filler for metal matrix composites [9,10]. However, Copper-graphene (Cu-Gr) metal composite was demonstrated to have a better conductance than Aluminum-graphene (Al-Gr) composite because the large lattice constant of Aluminum induces a significant lattice mismatch [11].

Copper-graphene composites, produced through many different methods, succeeded in enhancing the physical and mechanical properties of the composite [12]. Electroplated Cu-Gr coatings have already been produced to improve the pure copper characteristics; pulsed processes, in addition, can improve the morphological features of the composite coatings and, therefore, their performance. However, there is a lack of research and understanding of the pulsed electroplating process of copper-graphene films on aluminum substrate. Hwang et al. implemented a molecular-level mixing process to increase mechanical properties through excellent reduced Graphene Oxide (rGO) dispensability [13]. Cu-Gr anti-friction coatings were realized through a one-step procedure by Li et al. [14], or via a spark plasma sintering process by Chu et al. [15]. A hardness increment of about 40% was reached through a CVD process in [16], while a high-pressure torsion method allowed an improvement of the Cu-Gr interfacial bonding and, also, a hardness and the electrical conductivity increment in [17].

Electrodeposition is a simple and viable approach to produce a metal composite with enhanced mechanical and physical properties [18,19,20,21,22,23,24,25]. Compared to other methods, it allows the coating to cover large surfaces both reducing the cycle time and ensuring deposit purity [26]. Compared to the pure Cu film, Electrodeposited Cu-GO nanocomposite ones increased the thermal conductivity [27,28], while Cu-rGO/nanocomposite film showed higher electrical conductivity and corrosion resistance when applied as electrical contact material on copper foils [29]. Instead, the electroplated coating of copper and graphene nano-platelets increased the wear resistance of pure copper [30]. The process parameters of the Cu-Gr electroplating process on a copper substrate were adjusted by Song et al. to maximize the graphene co-deposition and, therefore, the coating performance [31,32].

During direct current (DC) electroplating, the diffusion layer forming around the cathode hinders the ions deposition limiting both the growth rate and the mechanical properties of the deposits. Adjusting the current density and varying the current over the time process allows for controlling the structure of the electrodeposits and their morphology [33,34,35]. In pulse and pulse-reverse electrodeposition, the current is applied in short pulses. In particular, during pulse electrodeposition the current is alternately switched off/on, while during pulse-reverse electrodeposition, anodic pulses follow cathodic pulses. Both methods have been demonstrated to enhance the deposition process control compared to the DC electroplating. Indeed, the switch on/off of the current dissipates the diffusion layer, whereas the reverse current reduces the hydrogen entrapment, residual stress and additive consumption [36,37]. Additionally, the inclusion of the particles within the metal matrix, driven by Guglielmi’s model, is affected by the operational parameters of the pulsed process [38].

Despite the large number of publications on pulse-reverse electroplating, there is still limited information regarding the effect of microstructure on the properties of the deposit [39]. Additionally, very few studies investigate the electroplating of copper-graphene on aluminum substrates to study the effect of pulse parameters. Hence, the present investigation presents the development of a Cu-Gr composite coating by using a pulse-reverse electrodeposition on an AA1370 aluminum wire. The graphene adopted as reinforcement was in the form of nanoplates (GnP). To study the effects of pulse parameters, such as the current density, frequency and duty cycle, a 2^3^-factorial plan was developed and tested. The analysis of Variance ANOVA was adopted to evaluate their influence on the morphology and electrical conductivity resistance of the coated wires.

## 2. Materials and Methods

Aluminum AA1370 was used as the substrate material. The samples, 1000 mm in length, were cut from a wire with a diameter of 2 mm, produced by drawing. The alloy belongs to the 1XXX series, characterized by high aluminum content; its chemical composition is shown in Table 1. The sample surface was sandblasted before the electroplating process to remove oil residues and oxide layers while obtaining a rough surface. The latter is mandatory to obtain a good adhesion between the substrate and the deposit [40]. The sandblasting process was performed using corundum spheres (180–212 μm), at high pressure (6 bar), for 8 s, according to [41]. Due to the high oxidation rate of the substrate material, the electrodeposition was performed immediately after the sandblasting process.

The electrodeposition process consists of two steps: in the first step, a thin layer of pure copper is electrodeposited on the substrate to increase the chemical affinity with the composite coating; in the second one, copper and graphene are co-electrodeposited on the copper layer to obtain a multilayered coating of 15 µm in thickness. Graphene nanoplates (GnP), provided by Nanesa Srl, were used as the second phase; such a choice was motivated by their low purchase costs (compared to nanotubes), excellent thermal and electrical conductivity. The main properties of GnPs are shown in Table 2.

The process steps differ in the galvanic bath, as reported in Table 3 and Table 4.

**Figure 1:**
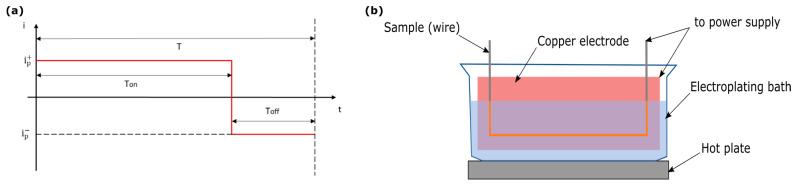
(**a**) Square wave for the AC galvanic process; (**b**) schematic of the electroplating process.

**Figure 2:**
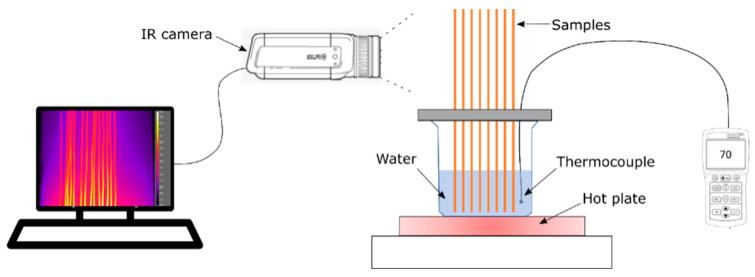
Schematic of the thermal measurement system.

The pure copper layer was applied through DC electrodeposition, whereas the Cu-GnP layer was electrodeposited by using an AC source and a squared waveform as represented in Figure 1. The wire to be coated was connected to the negative power supply; the sacrificial electrode was composed of two pure copper plates connected to each other to the positive power supply. The bath was kept in agitation by a recirculating system that maintains process temperature. The schematic representation of the experimental setup is illustrated in Figure 2. The first step was performed at room temperature, while the second one at 60 °C. 

The main characteristic parameters of pulsed electrodeposition are defined as follows:

- On-time (T_ON_)
- Off-time (T_OFF_)
- Peak current density (i_P_)

The forward current $i_{p}^{+}$(positive) is applied during the On-time, while the reverse current $i_{p}^{-}$(negative) is provided during the Off-time. Their values are equal in modulus so that the amplitude of the resulting waveform is symmetric around zero. The duty cycle is defined as the ratio between *T_ON_* and the wave period (*T*), as reported in Equation (1):

(1) $$Duty=\frac{T_{ON}}{T_{ON}+T_{OFF}}=T_{ON}\frac{1}{T}=T_{ON}*f\;\left[\%\right]$$

where *f* is the pulse frequency.

The average current density (*i_a_*) is defined as follows:

(2) $$i_{a}=i_{P}{}_{p}^{+}Duty+i_{P}{}_{p}^{-}\left(1-D\right)=i_{P}{}_{p}^{+}\left(2D-1\right)\;[A/{\mathrm{dm}}^{2}]$$

The deposition rate can be estimated from the DC electrodeposition at current density *i_a_*.

Since many combinations of process parameters are possible in pulsed electrodeposition, it was decided to use a statistical approach in order to reduce the number of trials. Based on preliminary experiences, a 2^3^ factorial plan was developed and tested. The following electroplating parameters were varied: the current density, the frequency, and the duty cycle. The control factors (i.e., the electroplating parameters) and their levels are reported in Table 5. Table 6 summarizes the different test scenarios.

The deposition time was evaluated through the Faraday’s law, considering a layer of pure copper of 5 µm and a Cu-Gr functionalized coating of 10 µm. The electrodeposition was performed by using an AC source (BOP 50-2D by KEPCO), controlled through National Instruments Data Acquisition System (DAQ) and LabView software.

The coating characterization was performed by means of a Scanning Electron Microscope (SEM) to highlight the morphology, and roughness analysis was carried out through a profilometer (Talysurf CLI 2000) to acquire the roughness parameters of the coatings.

The wires’ electrical resistance was measured using a resistance meter (DC series 2840, B&K Precision Corporation, Yorba Linda, CA, USA) equipped with Kelvin test leads with a 4-wire terminal. The measurement was performed on 300 mm-in-length portions of the wire; the procedure was repeated for each of the three portions of the wire to ensure the repeatability of the results. The electrical resistivity was calculated according to Equation (3):

(3) $$\rho =\frac{R\cdot A\;}{l}\;[m\Omega \;\mathrm{mm}]$$

with *R* representing the electrical resistance, *A* the cross-sectional area of the conductor and *l* the length of the wire portion. The cross-section evaluation was carried out by considering a theoretical coating thickness of 15 µm.

The thermal properties of the coatings were assessed through heating tests, the setup of which is represented in Figure 2. The wires were blackened and placed inside a beaker containing 500 mL of water heated to 60, 70, 80 and 90 °C, and the water was kept in agitation by a magnetic stirrer. The temperature of the wires was acquired using an infrared camera (A655SC, FLIR). As a consequence, the coated wire that reaches the higher temperature (given the distance from the free surface of the hot water) is the one that shows the best thermal performance. The composite coatings’ electrical and thermal performance was determined as a percentage increment over the aluminum one.

The effect of process parameters (control factors) on the morphology and electrical resistivity (response variables) of the coated wires was assessed through the ANOVA analysis by using the statistical software Minitab 18. The analysis was conducted at 95% of confidence level, the *p*-value < 0.05 was selected to determinate if the control factors (or their combinations) are statistically significant. Before the ANOVA, the residues were checked through the graphical analysis in order to confirm the underlying hypotheses. Then, the main effect plots were used to investigate the relation between the control factors and the coating performance. The interaction plots were additionally reported to highlight the synergic or anti-synergic effect due to the combination of control factors. 

## 3. Results and Discussion

**Figure 3:**
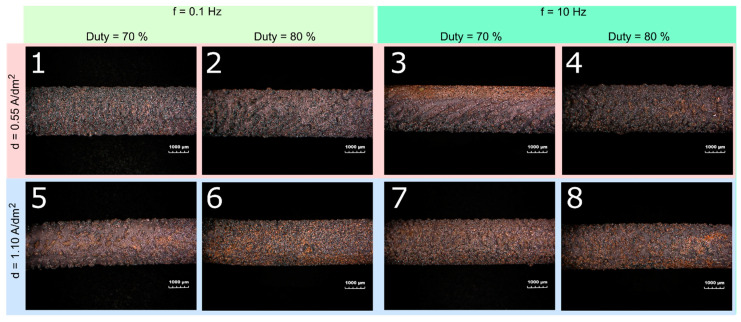
Morphology of the Cu-GnP composite coatings: the number in white represents the test scenario (see Table 6).

**Figure 4:**
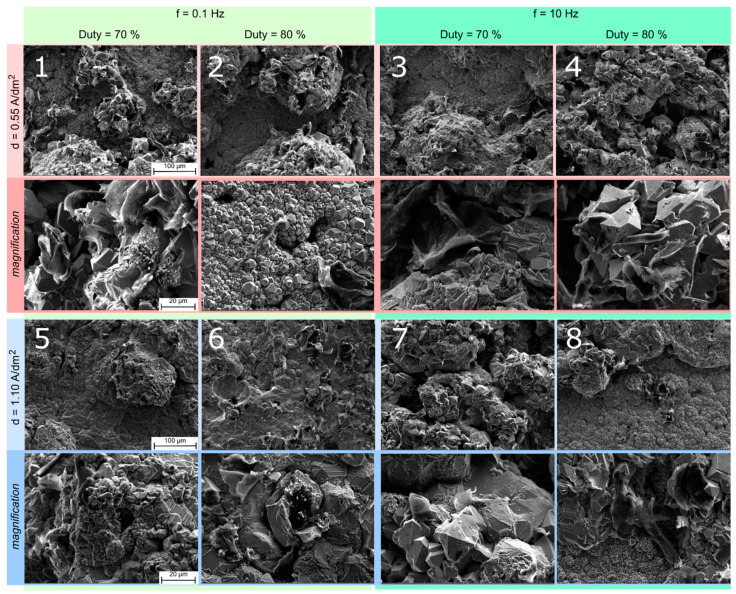
SEM images of the Cu-GnP composite coatings: the number in white represents the test scenario (see Table 6).

Figure 3 shows the morphology of the electroplated coatings, and Figure 4 its SEM magnification. In Figure 3, the coatings show a rough surface due to the presence of the graphene nanoplates confirmed by SEM images. The morphology is affected by the electroplating parameters: more in detail, at low frequency (f = 0.1 Hz), the duty cycle does not seem to exert a significant influence on the morphology; conversely, the latter is affected by the current density. The coatings electrodeposited at low density (d = 0.55 A/dm^2^) appear darker and more uniform, while the ones electrodeposited at high current density (d = 1.10 A/dm^2^) show dark spots and lighter areas.

The same considerations can be drawn at high frequency (f = 10 Hz). Additionally, the effect of the duty cycle at low current density is quite evident: the higher the duty cycle, the more the clusters of graphene on the surface of the coating.

The microstructure of the coatings is shown in Figure 4. Graphene nanoplates are recognizable within the copper matrix. The coating obtained at a low current density (d = 0.55 A/dm^2^) exhibits a higher graphene content, which, acting as a preferential growth site for copper reduction, promotes the formation of clusters. The low frequency (f = 0.1 Hz), however, inhibits the growth of copper grains. Under these conditions, graphene flakes cannot be completely embedded into the metal matrix: the coating presents a smooth surface characterized by the absence of clusters. At low duty cycle, but high frequency and current density, the coating shows a mixed morphology, alternating fine and large grains. The clusters interspersed with empty spaces results in a loose layer. At high duty cycle, the coating structure appears more compact and smooth, highlighting the graphene anchored to the surface.

**Figure 5:**
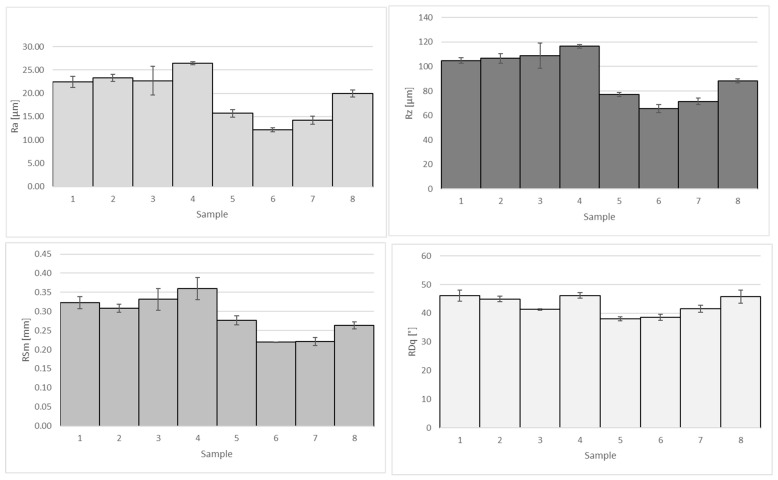
Roughens parameters of the composite coatings produced with different scenarios.

Figure 5 depicts the main roughness parameters of the composite coatings. The functionalized coatings present Ra values in the range of 11.91–26.19 µm against 1.42 µm of pure copper. This is ascribable to graphene, which, providing preferential sites for copper reduction, favors the formation of the clusters highlighted by SEM analysis.

The measured values of Ra and Rz follow the same trend; in fact, the coatings produced at low current density exhibit the highest roughness parameters (samples 1–4). Since the surface roughness and the amount of graphene are correlated, the low current density induces a higher incorporation of GnPs within the copper matrix. On the other hand, the high current density encourages the nucleation of copper grains and the consequent formation of adhesion points. Their growth is blocked by the surrounding grains, effectively limiting the height or reducing the space between contiguous peaks. The slope of the roughness profile is substantially constant under all tested conditions since the graphene particles incorporated into the growing deposit possess similar sizes.

In addition, ANOVA was performed to evaluate the effects of the process parameters. The results in terms of P-value and F-value are reported in Table 7, where significant control factors and interactions are highlighted in bold. The higher the F-value, the higher the statistical significance of the control factor.

**Figure 6:**
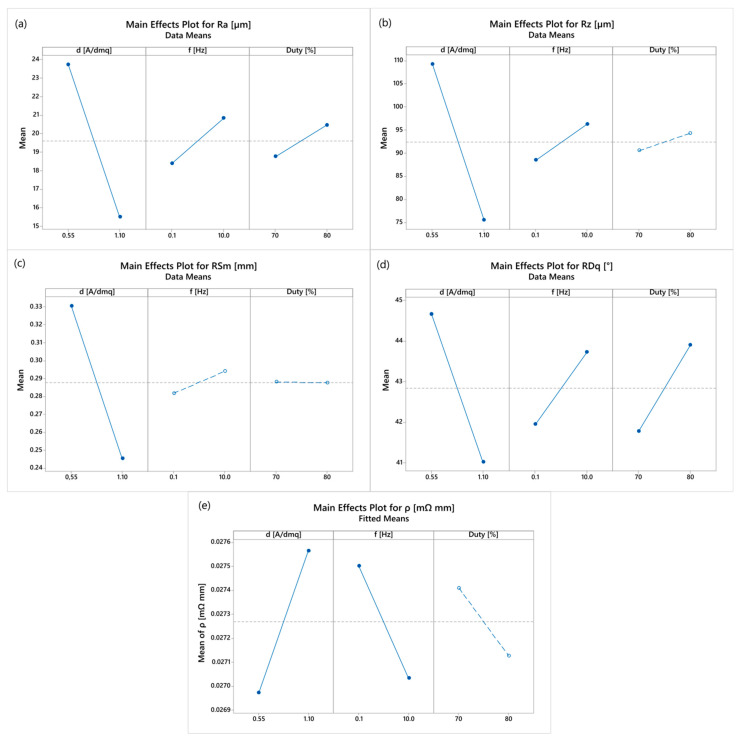
Main effect plots for: (**a**) Ra, (**b**) Rz, (**c**) RSm (**d**) RDq and (**e**) *ρ*; not statistically significant plots are in dashed lines.

In order to detect how the significant control factors affect the roughness parameters and electrical resistivity of the coatings, the main effect plots are reported. In Figure 6, the plots illustrating not statistically significant control factors (whose *p*-value does not exceed 0.05) are highlighted in dashed lines. Figure 6a shows a decrease in the arithmetic average roughness of the coatings as the current density increases. This result can be explained by considering that low current densities promote the growth of the crystalline grains forming the deposit and inclusion of graphene within the matrix. These phenomena are responsible for the increment in surface roughness. Conversely, Ra increases with increasing both the pulse frequency and duty cycle. The increase in pulse frequency corresponds to a decrement in the wave period. Accordingly, both T_ON_ and T_OFF_ are reduced. The pulse duration influences the selective deposition/dissolution of the deposit: during the OFF-time of the reverse pulse electrodeposition (RPP), copper is dissolved into the electrolyte selectively from the areas of higher electrical charge density localized on the surface of the deposit. Therefore, the high electrical conductivity of graphene can result in localized dissolution of copper from the area surrounding the clusters and consequent increase in the surface roughness. In addition, charge and discharge times become more relevant when the wave period is reduced. However, the effect of pulse frequency on the arithmetic average roughness is less relevant than that caused by the current density, as confirmed by the lower F-value. An increase in Ra is also observed as the duty cycle increases, because the process tends towards the DC electrodeposition having the same $i_{a}$. The same considerations can be drawn in relation to the other roughness parameters. 

Regarding the electrical performance of the coatings, the higher the current density, the greater the electrical resistivity of the material. This can be explained by considering the higher amount of graphene that is embedded in the metal matrix during co-electrodeposition, indicated by the high surface roughness. This result is also confirmed by the SEM observations reported above.

**Figure 7:**
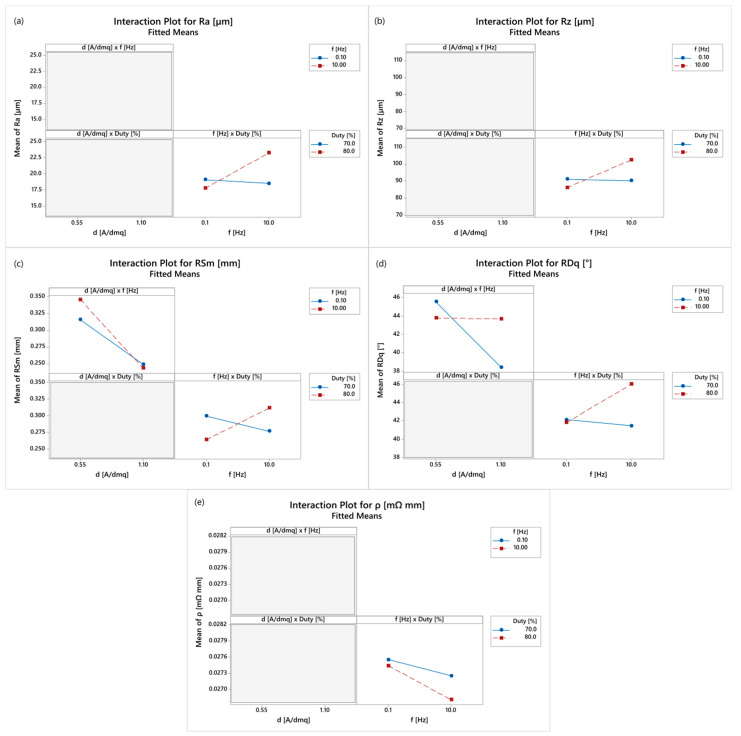
Interaction plots: (**a**) Ra, (**b**) Rz, (**c**) RSm, (**d**) RDq and (**e**) *ρ*; not statistically significant interactions are masked or not reported.

The interactions plots are shown in Figure 7. As regards Ra, a significant interaction is found between the pulse frequency and duty cycle. The combination of high pulse frequency and duty cycle causes an increase in surface roughness. The duty cycle is also defined by the product of the On-time and pulse frequency. Given the pulse frequency, an increase in duty cycle corresponds to an increase in T_ON_ that has a stronger effect at high pulse frequency when the wave period is reduced. 

Regarding Rz, the same considerations apply as above for Ra. The peaks observed on the surface of the coatings can be related to the micrometric size of graphene particles differently aligned in the copper matrix. These areas become preferential sites for the growth of the copper deposit, further increasing the surface roughness.

Concerning RSm, the combination of low duty cycle and low frequency produces an increase in the spacing of the elements in the roughness profile. At low pulse frequency, an increase in T_OFF_ significantly affects the surface roughness at low duty cycle when the On-time is reduced. Since the F-value is very low, the interaction of current density and pulse frequency can be neglected. The same considerations drawn for Ra and RSm apply to RDq, but the interaction of current density and pulse frequency is not negligible. However, in the interaction plots, the effect of the pulse frequency is reduced when the low current density is applied. 

**Figure 8:**
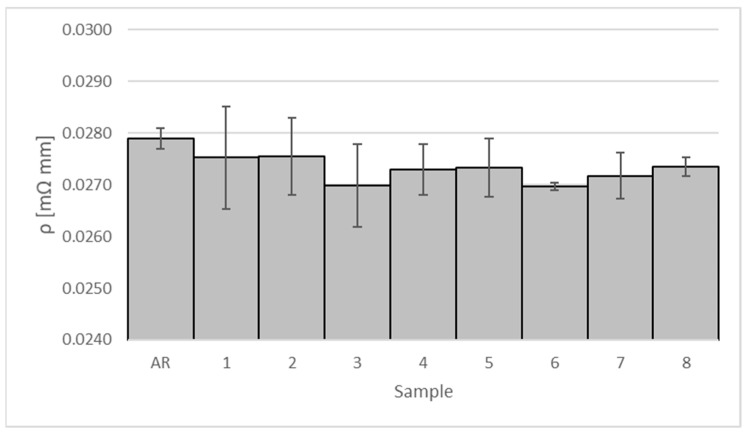
Electrical resistivity of the coated samples.

The electrical resistivity of the coated samples and its percentage reduction with respect to the as-received one are shown in Table 8 and Figure 8, respectively.

**Figure 9:**
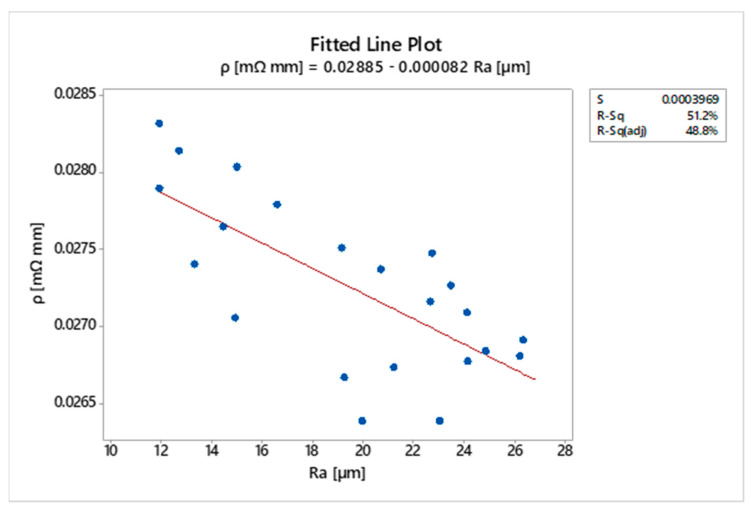
Regression analysis: *ρ* vs. Ra (*p*-value = 0.000).

The greatest improvement of the electrical resistivity (of about 3.3%) is found in scenarios 3 and 6. However, the roughness profile of the coatings exhibits peaks of ~100 µm in height affecting the contact resistance. The clamps of the 4-wire terminals of the measuring instrument do not adhere perfectly to the asperities on the surface of the coatings. Therefore, it is worth noting the negative correlation between Ra and the electrical resistivity of the coated samples in Figure 9. Despite a reliability index (R-sq) of about 50%, the *p*-value of 0.000 indicates a strong linear relationship between surface roughness and electrical performance. This trend confirms the considerations given above about the main effect plots of the electrical resistivity. The low value of R-sq is probably due to unusual observations affecting the correlation. These are ascribable to the high surface roughness, which increases the contact resistance.

**Figure 10:**
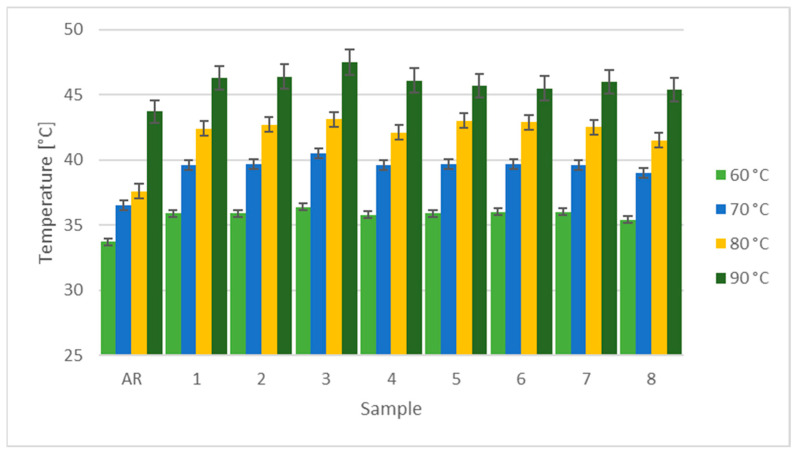
Temperature reached by the wires during the thermal tests.

Thermal characterization of the coated samples is not affected by their contact resistance, since they are immersed in water. The performance among scenarios is homogeneous, as illustrated in Figure 10. The Cu-GnP composite coatings achieve a relevant improvement in thermal properties over the substrate material.

In addition, there is a match for the best-case scenario in both fields represented by sample 3. It shows the highest temperature of around 47 °C. It is clear, therefore, that thermoelectric performance increases significantly following the application of such a functionalized coating. Although the high surface roughness indicates a greater amount of graphene, it also reduces the electrical performance by increasing the contact resistance. However, the quality of the coatings is confirmed by the thermal characterization, which shows increased conductivity under all scenarios if compared to the untreated substrate.

## 4. Conclusions

In the present paper, Cu-Gr composite coatings were developed by using pulse-reverse current electrodeposition on AA1370 aluminum wires of 1000 mm in length and diameter of 2 mm. In order to study the effects of current density, pulse frequency and duty cycle, a 2^3^ factorial plan was developed and tested. Based on the experimental results, the following findings can be drawn:

- The Cu-GnP composite coatings exhibit low surface roughness (Ra of 5.63 µm, RSm of 0.12 mm and RDq of 30.72°).
- At high current density (d = 1.1 A/dm^2^), the nucleation process is favored over the growth, resulting in smoother and more compact coatings; the surface roughness increases when increasing the pulse frequency and duty cycle because the “noise” increases and the process tends towards DC electrodeposition.
- SEM investigations show that all test scenarios provide good results in terms of coating density and compactness, amount of graphene and flakes well incorporated within the microstructure of the copper deposits.
- The coated samples show lower electrical resistivity (up to −3.4%) than the uncoated aluminum substrate.
- The coated samples exhibit a percentage increase in temperature compared to the aluminum wire of about 10%.

## Figures and Tables

**Table 1:** AA1370 composition.

Elements	Wt%
Aluminum	≥99.70
Boron	≤0.02
Chromium	≤0.01
Copper	≤0.02
Gallium	≤0.03
Iron	≤0.25
Magnesium	≤0.02
Manganese	≤0.01
Silicon	≤0.10
Zinc	≤0.04

**Table 2:** GnP features.

Properties	Value
Carbon content	>97%_wt_
C:O ratio	49:1
Flake average thickness	8 nm
Flake lateral dimension	96 µm
Density	0.07 g/cm^3^
Specific surface area	56 m^2^/g
Electrical resistivity	0.001 Ω·cm
Thermal conductivity	1000 W/m·K

**Table 3:** Galvanic bath for pure copper deposition.

Components	Concentration
Copper sulphate hexahydrate	170 g/L
Sulfuric acid	60 g/L
Copper chloride	50 p.p.m.

**Table 4:** Galvanic bath for copper-graphene co-deposition.

Components	Concentration
Copper sulphate hexahydrate	170 g/L
Copper chloride	50 p.p.m.
GnP	1 g/L

**Table 5:** Control factors and their levels.

Control Factors	Symbols	Levels
Current density	d [A/dm^2^]	0.55–1.1
Pulse frequency	f [Hz]	0.1–10
Duty cycle	Duty [%]	70–80

**Table 6:** Test scenarios and their labels.

Sample	d [A/dm^2^]	f [Hz]	Duty [%]
1	0.55	0.1	70
2	0.55	0.1	80
3	0.55	10	70
4	0.55	10	80
5	1.1	0.1	70
6	1.1	0.1	80
7	1.1	10	70
8	1.1	10	80

**Table 7:** Anova results in terms of *p*-value and F-value.

Source	Ra		Rz		RSm		RDq		*ρ*	
	F-Value	*p*-Value	F-Value	*p*-Value	F-Value	*p*-Value	F-Value	*p*-Value	F-Value	*p*-Value
d [A/dm^2^]	162.49	**0.000**	233.72	**0.000**	126.22	**0.000**	45.03	**0.000**	15.06	**0.001**
f [Hz]	14.18	**0.002**	12.54	**0.003**	2.65	0.122	10.67	**0.005**	9.46	**0.008**
Duty [%]	6.88	**0.018**	2.80	0.112	0.00	0.965	15.12	**0.001**	3.41	0.085
d [A/dm^2^] × f [Hz]	1.24	0.281	0.14	0.713	5.53	**0.031**	42.47	**0.000**	9.90	**0.007**
d [A/dm^2^] × Duty [%]	0.83	0.375	0.25	0.620	0.98	0.336	0.22	0.648	0.27	0.614
f [Hz] × Duty [%]	22.44	**0.000**	15.32	**0.001**	21.72	**0.000**	19.93	**0.000**	1.18	0.294
R-sq [%]	92.45		93.97		90.24		88.70		70.81	

**Table 8:** Electric resistivity of the coated samples and their percentage reduction compared to the as-received one (AR).

Sample	d [A/dm^2^]	f [Hz]	Duty [%]	*ρ* [mΩ mm]	St. Dev. [mΩ mm]	Reduction [%]
AR	-	-	-	0.0279	0.0002	0.00
1	0.55	0.1	70	0.0275	0.0010	−1.36
2	0.55	0.1	80	0.0275	0.0007	−1.27
3	0.55	10	70	0.0270	0.0008	−3.29
4	0.55	10	80	0.0273	0.0005	−2.20
5	1.1	0.1	70	0.0273	0.0006	−2.03
6	1.1	0.1	80	0.0270	0.0001	−3.33
7	1.1	10	70	0.0272	0.0004	−2.62
8	1.1	10	80	0.0273	0.0002	−1.99

## Data Availability

The data are not publicly available due to information used for another publication.
